# High rates of viral suppression in pregnancy drop postpartum in South African women on tenofovir‐lamivudine‐dolutegravir: a prospective cohort study

**DOI:** 10.1002/jia2.70044

**Published:** 2025-10-23

**Authors:** Elaine J. Abrams, Jennifer Jao, Elton Mukonda, Hlengiwe P. Madlala, Sandisiwe Matyseni, Allison Zerbe, Justine Legbedze, Landon Myer

**Affiliations:** ^1^ ICAP at Columbia University, Mailman School of Public Health Columbia University New York New York USA; ^2^ Department of Epidemiology, Mailman School of Public Health Columbia University New York New York USA; ^3^ Department of Pediatrics, Vagelos College of Physicians & Surgeons Columbia University New York New York USA; ^4^ Department of Pediatrics and Department of Medicine Northwestern University Feinberg School of Medicine Chicago Illinois USA; ^5^ Division of Epidemiology and Biostatistics, School of Public Health University of Cape Town Cape Town South Africa

**Keywords:** antiretroviral therapy, dolutegravir, HIV, pregnancy, postpartum, viral load

## Abstract

**Introduction:**

Achieving and maintaining viral suppression (VS) during pregnancy and breastfeeding is central to preventing vertical transmission and optimizing maternal health. High rates of VS have been demonstrated among adult and paediatric populations receiving tenofovir‐lamivudine‐dolutegravir (TLD), but VS and viraemia among pregnant and postpartum women with HIV (WHIV) in high‐burden settings have not been well‐documented.

**Methods:**

Between September 2021 and December 2023, pregnant WHIV, ≤18 weeks gestation, were enrolled in antenatal care (ANC) and followed postpartum in Cape Town, South Africa. WHIV received HIV care in routine health services and continued, switched to or initiated TLD at ANC entry. VS was defined as viral load (VL) <50 copies/ml; viraemic episodes (VEs) were categorized as major (>1000 copies/ml) or minor (50−1000 copies/ml). Mixed‐effects Poisson regression models were fit to assess factors associated with major VE risk.

**Results:**

Among 763 WHIV with ≥1 VL, median age was 30 years (interquartile range [IQR] 25−34) and median gestation was 14 weeks at enrolment (IQR 11−17); 89% were on antiretroviral therapy, including 74% on TLD. Overall 99% achieved ≥1 VL<50 copies/ml: 73% sustained VS through 48 weeks postpartum, with 16% having ≥1 minor VE and 15% ≥1 major VE. At enrolment, 77% of VL measures were <50 copies/ml, increasing to >90% during pregnancy through 12 weeks postpartum and declining to 81% by 24 weeks postpartum. In multivariable analysis, each additional year of age conferred a 6% (95% confidence interval [CI] 0.89, 0.98, *p* = 0.006) lower risk of subsequent major VE after achieving VS. WHIV with viraemia (50−1000 copies/ml) at enrolment were 3.6 (95% CI 1.94, 6.70, *p*<0.001) times more likely to have a subsequent major VE, whereas CD4+>500 cells/mm lowered major VE risk by 53% (95% CI 0.32, 0.89, *p* = 0.016).

**Conclusions:**

High rates of VS were maintained during pregnancy and early postpartum, but substantial viraemia emerged by 24 weeks postpartum, jeopardizing maternal and child health outcomes. These unique data provide further impetus to explore innovative approaches to supporting adherence among WHIV during the postpartum period.

## 1 INTRODUCTION

Over the last 5 years, most low‐ and middle‐income countries (LMICs) have successfully scaled up integrase inhibitor‐based antiretroviral therapy (ART) [1]. It is estimated that 85−90% of individuals living with HIV on ART in LMICs are currently receiving a dolutegravir (DTG)‐containing regimen, primarily tenofovir + lamivudine + DTG (TLD) [2]. Early evidence suggests high rates of sustained viral suppression (VS) with TLD among adults and children, notably higher than achieved with the previous first‐line non‐nucleoside‐based regimen tenofovir + lamivudine + efavirenz (TLE) [3, 4, 5]. Two clinical trials reported high rates of VS (93−95%) among pregnant and breastfeeding women with HIV (WHIV) randomized to receive TLD, but virologic outcomes among pregnant and postpartum women in routine HIV care have received little attention [6, 7, 8, 9].

Historically, in South Africa and other high HIV prevalence countries, high rates of VS have been demonstrated during pregnancy, among women on ART prior to pregnancy, as well as those initiating treatment during pregnancy [8−10]. The postpartum period, however, has been identified as highly vulnerable to loss of viral control [10, 11, 12, 13, 14, 15]. In a cohort of 523 South African pregnant women initiating TLE during pregnancy and achieving VS, 22% experienced at least one viraemic episode (VE) with viral load (VL) >1000 copies(cps)/ml over a median follow‐up of 322 days [16]. Younger age, ART initiation in the third trimester and previous disengagement from care were identified as risk factors for loss of VS. Postpartum viraemia places the breastfeeding infant at risk for HIV acquisition and jeopardizes maternal health outcomes; in South Africa, over 50% of new paediatric HIV acquisitions occur during breastfeeding [17, 18].

We examined patterns and factors associated with VS and viraemia among pregnant and postpartum WHIV receiving TLD in routine health services enrolled in the Obesogenic oRigins of maternal and Child metabolic Health Involving Dolutegravir (ORCHID) study in Cape Town, South Africa [19].

## 2 METHODS

ORCHID is a prospective observational cohort study (NCT 04991402) investigating associations between DTG, weight gain and metabolic health outcomes among WHIV and their children. The design and methods have been described previously [19]. In brief, between September 2021 and December 2023, we enrolled 1920 pregnant women, 804 WHIV and 1116 HIV‐seronegative women, ≥ 16 years of age and ≤18 weeks gestational age, for follow‐up through 24 months postpartum. Potentially eligible women were identified at their first antenatal care (ANC) visit at primary care facilities located in the Klipfontein‐Mitchell's Plain region in Cape Town, and those who were interested in study participation were referred to a community‐based research site at the Gugulethu Community Health Centre, where enrolment and all study visits and procedures are conducted. ART initiation and management took place as part of routine healthcare services. TLD was the recommended ART first‐line regimen for adults with HIV, including pregnant and breastfeeding WHIV [20, 21].

In parallel to routine HIV and ANC services, participants attend up to 10 study visits, including three antenatal and seven postnatal visits. At each visit, participants complete study measures, including questionnaires collecting demographic and clinical information, as well as specimen collection. Separate from routine VL monitoring, a venous blood sample was collected at enrolment, trimesters two (T2, 24−28 weeks) and three (T3, 32−34 weeks), and every 6−12 weeks postpartum for de‐identified batched VL testing using the Abbott Alinity m HIV‐1 assay (Abbott Laboratories, Chicago, Illinois) conducted by the South African National Health Laboratory Services. De‐identification took place through the use of a participant identification number generated separately from patient clinical folder numbers or other unique identifiers, with an access‐controlled identification log used to link participant data during analysis.

For this analysis, we included all WHIV with an enrolment VL and ≥1 additional VL measurement through 80 weeks of observation reported between September 2021 and September 2024. We defined VS as <50 cps/ml and VEs as major (>1000 cps/ml) or minor (50−1000 cps/ml). We examined patterns of viraemia among all WHIV and categorized women by TLD exposure status at enrolment: WHIV naïve to ART initiating TLD (Initiators); women on TLE switching to TLD (Switchers); women receiving TLD (Continuers). Of note, among the WHIV in the Switcher group, VL at enrolment is the closest VL to the time of transition from TLE to TLD among the Switchers. We describe the proportion of samples with VS, major and minor VE at each study visit, as well as the distribution of VLs over time in women with VS at enrolment. Overall group trends were generated using locally weighted scatterplot smoothing. The analysis of incident VE was restricted to women who achieved VL<50 cps/ml and had subsequent VL testing. For this, mixed‐effects Poisson regression models were fitted to assess factors associated with the risk of major VE. All models included random intercepts for each participant, with other covariates included as fixed effects. Covariate selection was based on existing understandings of causal associations involving viraemia in this population. Results are expressed as incidence rate ratios with 95% confidence intervals (CIs). All analyses were conducted using R version 4.4.1 (R Core Team, Vienna, Austria).

All women provided written consent prior at enrolment. The ORCHID study was reviewed and approved by the Faculty of Health Sciences Human Research Ethics Committee of the University of Cape Town (UCT‐HREC 709/2020), and Institutional Review Boards (IRBs) of Columbia University Irving Medical Center (AAAU3541) and the Ann & Robert H. Lurie Children's Hospital of Chicago (2021‐4725).

## 3 RESULTS

Of 804 pregnant WHIV enrolled in ORCHID, 763 (95%) were included in this analysis (*n* = 41 women with only enrolment VL were excluded). At enrolment, the median age was 30 years (IQR 25.6, 34.4), gestation 14 weeks (IQR 11.0, 17.0), VL was 19 cps/ml (IQR 19, 49) with 77% <50 cps/ml and CD4+ cell count 509 cells/mm^3^ (IQR 347, 642); most women (94%) reported completing primary education (Table 1). Overall, 676 (89%) WHIV were on ART at enrolment, including 561 (74%) who were already receiving TLD and continuing it (Continuers). A total of 202 (26%) pregnant WHIV initiated TLD immediately prior to or at enrolment, including 87 (11%) who were ART naïve (Initiators) and 115 (15%) on TLE who switched from TLE to TLD (Switchers). At enrolment, the overall median duration on ART was 1672 days, and the median duration on TLD was 241 days. At enrolment, the median duration on ART and TLD varied by group: median 9 days on ART and 9 days on TLD for Initiators; 2063 days on ART and 9 days on TLD for Switchers; 1827 days on ART and 416 days on TLD for Continuers. At enrolment, ART naïve WHIV were significantly younger compared to Switchers and Continuers, with higher median VLs and lower median CD4+ cell counts.

**Table 1:** Characteristics of study participants, pregnant women with HIV with an HIV viral load measure at enrolment, enrolled in the ORCHID cohort, September 2021 thru December 2023, Cape Town, South Africa

Characteristic	Overall, *N* = 763^a^	Initiators, *N* = 87^a^	Switchers, *N* = 115^a^	Continuers, *N* = 561^a^	*p*‐value^b^
**Age (years)**					<0.001
Median (IQR)	30.0 (25.6, 34.4)	26.6 (23.4, 31.0)	29.7 (25.6, 33.4)	30.8 (26.4, 35.0)	
Range	16.0−47.1	16.0−39.3	17.3−42.1	16.0−47.1	
**Gestational age (weeks)**					0.028
Median (IQR)	14.0 (11.0, 17.0)	13.0 (11.0, 15.5)	13.0 (10.0, 16.0)	14.0 (11.0, 17.0)	
Range	6.0−20.0	7.0−19.0	6.0−20.0	6.0−20.0	
**Gravidity**					<0.001
Primigravida	155 (20%)	35 (40%)	20 (17%)	100 (18%)	
Multigravida	608 (80%)	52 (60%)	95 (83%)	461 (82%)	
**Education**					>0.9
Any basic education	719 (94%)	82 (94%)	108 (94%)	529 (94%)	
Any post secondary education	44 (5.8%)	5 (5.7%)	7 (6.1%)	32 (5.7%)	
**Ever breastfed**					0.6
No	19 (2.7%)	3 (3.8%)	3 (2.8%)	13 (2.5%)	
Yes	679 (97%)	75 (96%)	103 (97%)	501 (97%)	
Unknown	65	9	9	47	
**Duration breastfeeding (weeks)**					0.2
Median (IQR)	19 (9, 41)	18 (9, 42)	20 (9, 44)	19 (9, 41)	
Min—Max	0−64	0−58	0−55	0−64	
Unknown	66	9	9	48	
**Viral load at enrolment (copies/ml)**					<0.001
Median (IQR)	19 (19, 49)	99 (19, 1026)	56 (19, 385)	19 (19, 19)	
Range	19−980,148	19−980,148	19−246,397	19−123,647	
**Viral load at enrolment (copies/ml)**					<0.001
<50	584 (77%)	31 (36%)	56 (49%)	497 (89%)	
50−1000	122 (16%)	34 (39%)	41 (36%)	47 (8.4%)	
>1000	57 (7.5%)	22 (25%)	18 (16%)	17 (3%)	
**CD4+ count at enrolment (cells/mm^3^)**					<0.001
Median (IQR)	509 (347, 642)	351 (263, 570)	378 (252, 588)	531 (400, 673)	
Range	16−3280	74−1029	30−1746	16−3280	
Unknown	24	2	4	18	
**ART status**					<0.001
ART initiators	87 (11%)	87 (100%)	0 (0%)	0 (0%)	
ART continuers	676 (89%)	0 (0%)	115 (100%)	561 (100%)	
**Duration on ART (days)**					<0.001
Median (IQR)	1672 (574, 2886)	9 (6, 14)	2063 (1420, 2881)	1827 (907, 3151)	
Range	2−9913	2−26	32−6400	29−9913	
**Duration on TLD (days)**					<0.001
Median (IQR)	241 (22, 608)	9 (6, 14)	9 (7, 14)	416 (174, 776)	
Range	0−1766	0−26	2−27	28−1766	
**Tests per WHIV**					0.3
Median (Q1, Q3)	6 (5, 7)	6 (4, 7)	6 (5, 7)	6 (5, 7)	
Range	2–7	2−7	2−7	2−7	
**Total person time (months)**	9675	1100	1523	7052	<0.001
**Viral load suppression (<50 copies/ml)**					<0.001
Suppressed^c^	752 (99%)	79 (91%)	115 (100%)	558 (99%)	
Never suppressed	11 (1.4%)	8 (9.2%)	0 (0%)	3 (0.5%)	
**Viral load suppression (<1000 copies/ml)**					0.001
Suppressed^c^	759 (99%)	84 (97%)	115 (100%)	560 (100%)	
Never suppressed	4 (0.5%)	3 (3.4%)	0 (0%)	1 (0.2%)	
**Number of elevated viral loads after first suppression (>50 copies/ml)**					<0.001
0	546 (73.0%)	54 (68%)	59 (51%)	433 (78%)	
1	107 (14%)	17 (22%)	26 (23%)	64 (11%)	
2	60 (8%)	3 (3.8%)	18 (16%)	39 (7%)	
3	23 (3.1%)	2 (2.5%)	7 (6.1%)	14 (2.5%)	
4	12 (1.6%)	3 (3.8%)	3 (2.6%)	6 (1.1%)	
5	4 (0.5%)	0 (0%)	2 (1.7%)	2 (0.4%)	
Unknown	11	8	0	3	
**Number of minor elevated viral loads after first suppression (50**−**1000 copies/ml)**					0.1
0	631 (84%)	68 (86%)	87 (76%)	476 (85%)	
1	93 (12%)	10 (13%)	25 (22%)	58 (10%)	
2	18 (2.4%)	1 (1.3%)	1 (0.9%)	16 (2.9%)	
3	8 (1.1%)	0 (0%)	2 (1.7%)	6 (1.1%)	
4	1 (0.1%)	0 (0%)	0 (0%)	1 (0.2%)	
5	1 (0.1%)	0 (0%)	0 (0%)	1 (0.2%)	
Unknown	11	8	0	3	
**Number of major elevated viral loads after first suppression (>1000 copies/ml)**					<0.001
0	636 (85%)	61 (77%)	79 (69%)	496 (89%)	
1	60 (8%)	12 (15%)	14 (12%)	34 (6.1%)	
2	36 (4.8%)	3 (3.8%)	13 (11.%)	20 (3.6%)	
3	10 (1.3%)	1 (1.3%)	4 (3.5%)	5 (0.9%)	
4	10 (1.3%)	2 (2.5%)	5 (4.3%)	3 (0.5%)	
Unknown	11	8	0	3	

Abbreviations: ART, antiretroviral therapy; IQR, interquartile range; TLD, tenofovir‐lamivudine‐dolutegravir; WHIV, women with HIV.

^a^ *N* (%).

^b^ Kruskal−Wallis rank sum test; Pearson's Chi‐squared test; Fisher's exact test.

^c^ Suppressed: Ever suppressed with one or more VL < 50 copies/ml.

**Figure 1:**
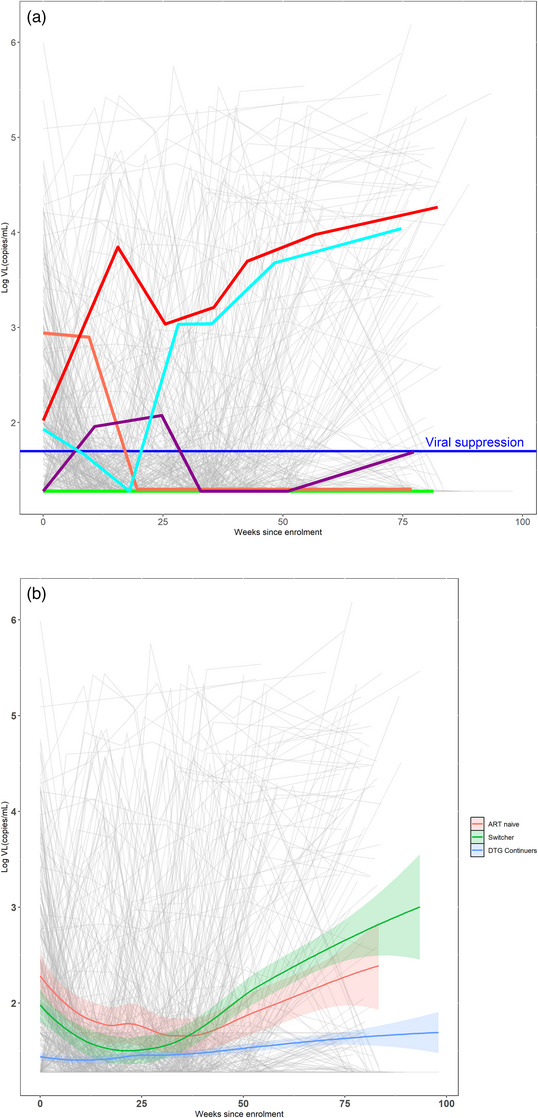
(a) Individual viral load (VL) trajectories for 763 women with HIV (in grey) from enrolment in pregnancy through 80 weeks of observation, ORCHID study, Cape Town, South Africa; Green represents women with HIV (WHIV) with viral suppression at enrolment or who achieved viral suppression during follow‐up and maintained viral suppression throughout follow‐up. Light blue represents WHIV with one or more minor viraemic episodes (VEs). Purple represents those WHIV with one or more major VE, and red includes WHIV who never achieved viral suppression. (b) Smoothed VL trends by enrolment ART status (Initiators, Switchers and Continuers).

A total of 9675 woman‐months of observation were accrued in the cohort; 58% of observations took place postpartum (5577 vs. 4099 woman‐months antepartum) and 4152 VL tests were conducted, median 6 tests per WHIV, range 2−7. During the postpartum period, 97% reported breastfeeding their infants. The median duration of breastfeeding through complete weaning was 19 weeks (IQR, 9, 41). Figure 1a shows individual viral trajectories for 763 individual WHIV from enrolment through the follow‐up period. Overall, 99% (752/763) of the cohort had ≥1 VL <50 cps/ml. Of these, 73% (546/752) sustained VS across the observation period, including 474 virally suppressed at enrolment and 72 achieving VS at a later time point. Sixteen percent had ≥1 minor VE and 15% had ≥1 major VE (Figure 1a and Table 1). Figure 1b describes trends in VL over time by ART group. Initiators were significantly more likely to never achieve VS (9%) compared with Switchers (0%) and Continuers (1%), *p*<0.001. Major VEs occurred among 23% of Initiators, 31% of Switchers and 11% of Continuers (Table 1).

At enrolment, 77% (584/763) of VL measures were <50 cps/ml (Figure 2a). The proportion of VL measures with <50 copies/ml increased from enrolment to 90% at T2 (24−28 weeks), T3 (32−34 weeks) and 6 weeks postpartum. The proportion with VS continued to decline to 89% and 81%, respectively, at 12 and 24 weeks postpartum. By 48 weeks, only 79% of measures were VS, whereas 17% (54/316) were >1000 cps/ml. While the pattern of VS from enrolment through 48 weeks postpartum was similar by enrolment ART status, the proportion of VEs varied by timing of TLD initiation (Figure 2b). The Continuer group had the highest proportion of suppressed VL measures across all study time points, ranging from 89% at enrolment to 88% at 48 weeks postpartum. As expected, VS was low at enrolment (36%) in the Initiator group, increasing thereafter but remaining <90% throughout pregnancy and the early postpartum period. By 24 weeks postpartum, 25% (13/53) of samples in the Initiator group were >1000 cps/ml. The Switcher group had higher levels of VS in pregnancy than the Initiators, but the increase in VEs was noted at 12 weeks postpartum, earlier in the Switcher group compared to 24 weeks for the two other groups.

Figure 2(a) Distribution of viral load (VL) results during pregnancy at enrolment, trimester 2 (24−28 weeks), trimester 3 (34−36 weeks), and 6, 12, 24 and 48 weeks postpartum among 763 women with HIV (WHIV). (b) Viral load distribution by antiretroviral therapy (ART) status at enrolment (currently receiving tenofovir+lamivudine+dolutegravir/TLD [Continuers], switching from tenofovir+lamivudine+dolutegravir/TLE to TLD [Switchers], newly initiating ART with TLD [Initiators]) among 763 WHIV, ORCHID study, Cape Town, South Africa.

**Figure:**
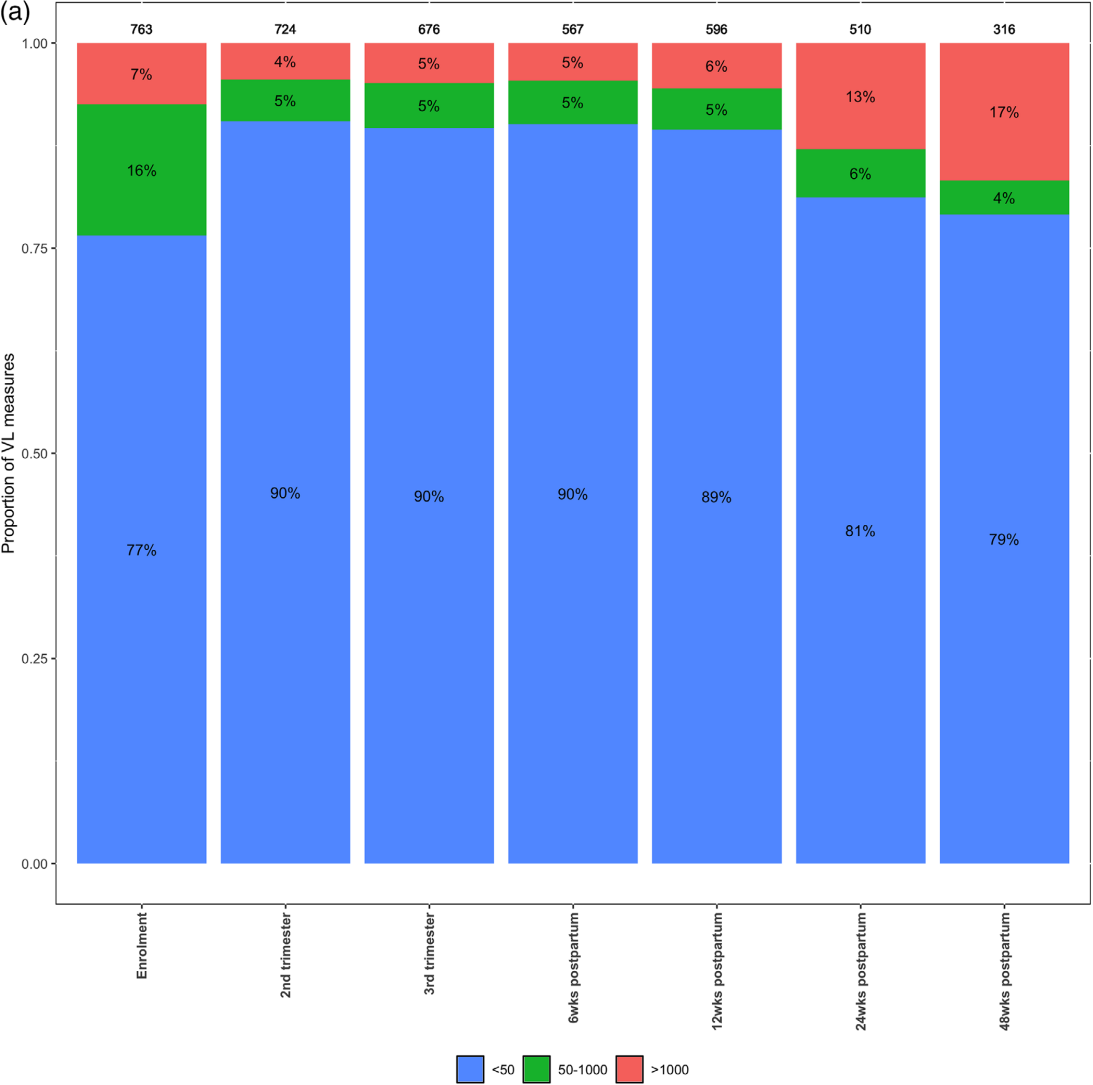

**Figure:**
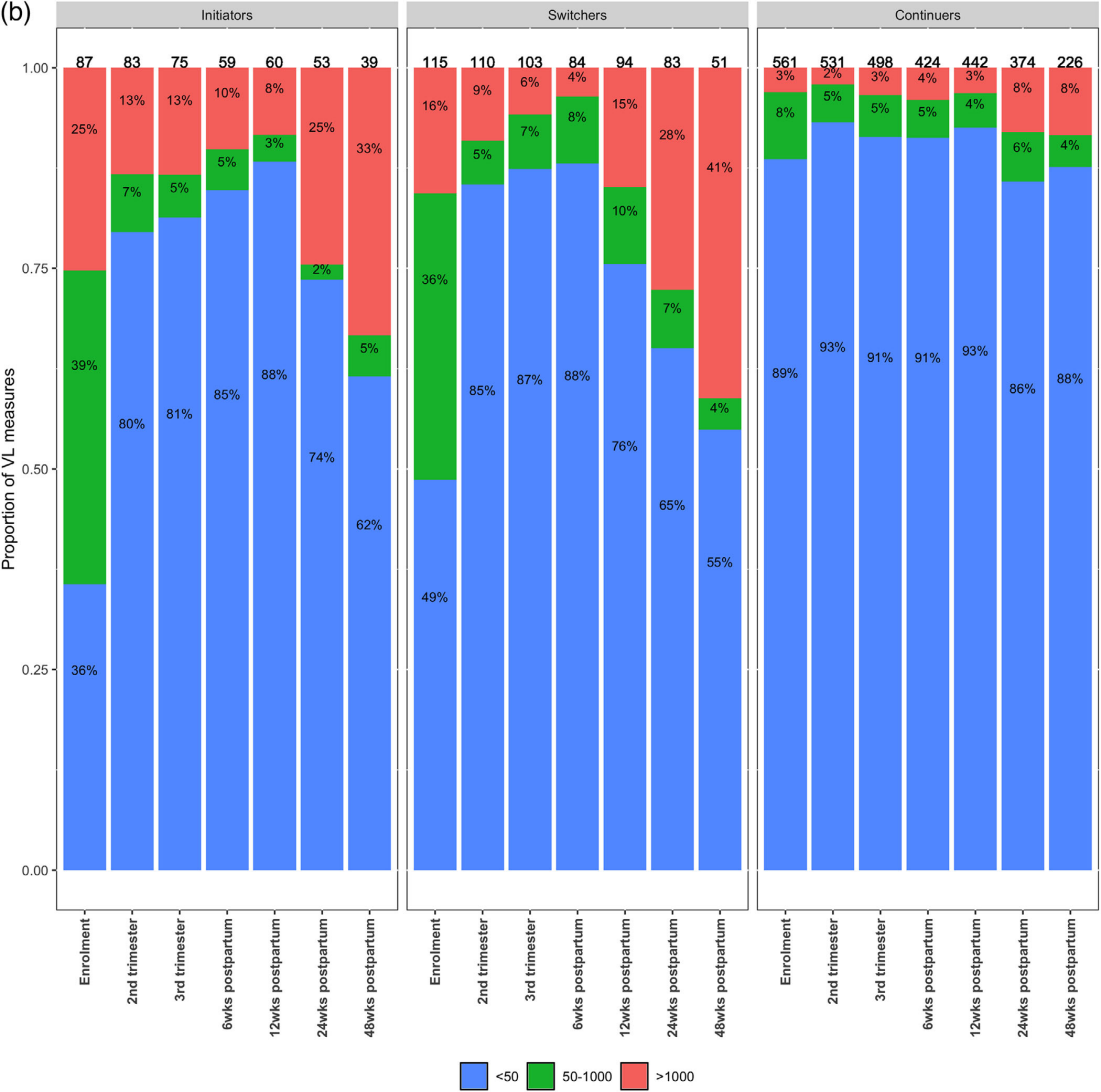

**Figure 3:**
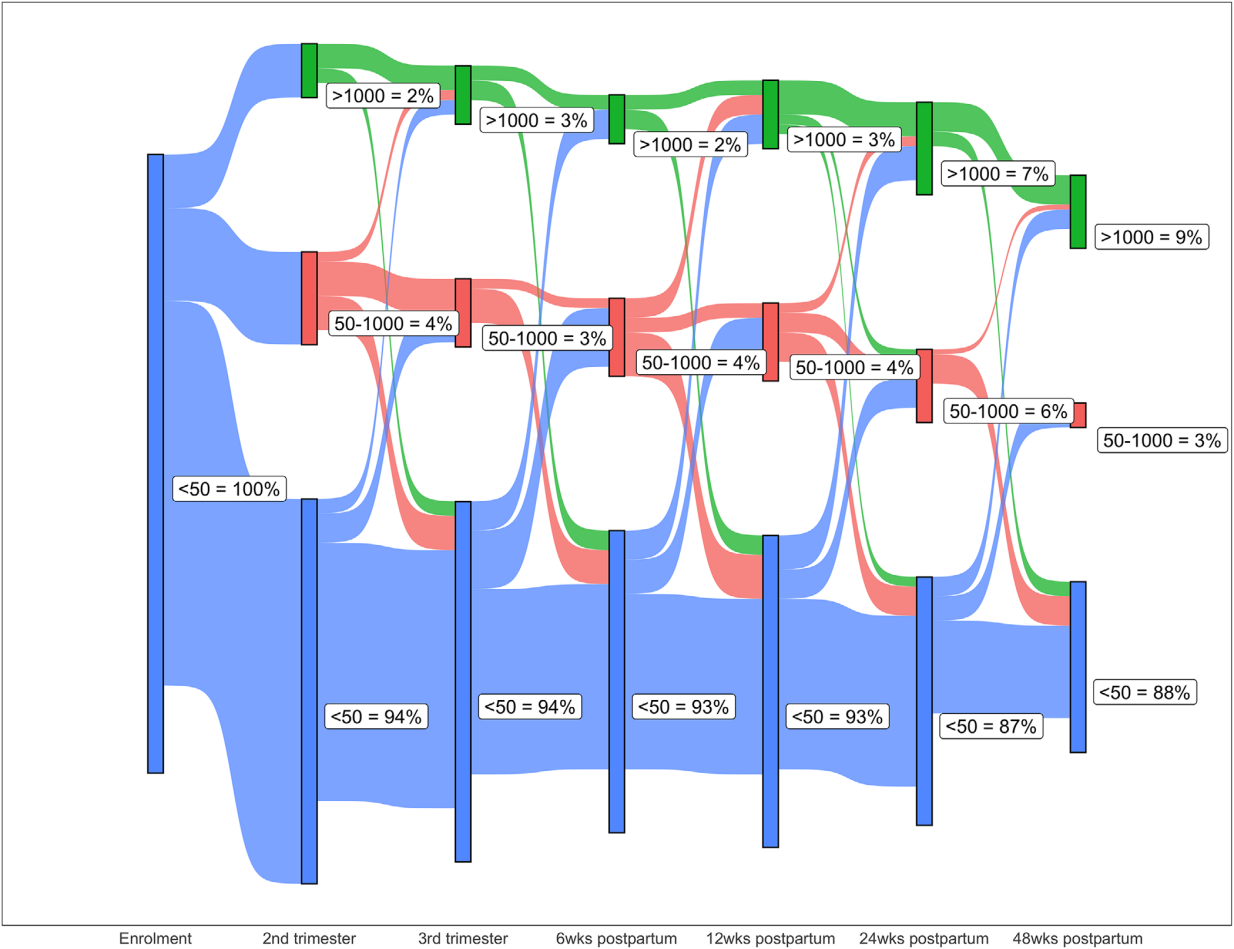
Plot of transitions between viral load (VL) categories across study visits during pregnancy through 48 weeks postpartum among women with HIV (WHIV), ORCHID study, Cape Town, South Africa. Blue represents VL <50 copies/ml, red represents VL 50−1000 copies/ml and green represents VL >1000 copies/ml.

In a sub‐analysis of 584 women with VL <50 cps/ml at enrolment, including 497 (85%) WHIV in the Continuer group, we found that most women (80%, *n* = 466) had no VEs through 48 weeks postpartum (Figure 3). Furthermore, among WHIV with measures at 48 weeks postpartum, 88% were <50 copies/ml. Overall, few women moved in and out of VS over time, with 3−6% having a minor VE and 2−9% a major VE at any study visit. Among this group of WHIV, the proportion of WHIV with minor and major VEs nearly doubled from 24 to 48 weeks postpartum. Overall, 52% (61/118) of WHIV regained VS after a VE.

In univariable analysis, restricting the cohort to WHIV who achieved VS, age, ART status, VL and CD4+ at enrolment were each associated with the risk of subsequent major VE (Table 2). In multivariable analysis, we found that each additional year of age was associated with a 6% (95% CI 0.89, 0.98, *p* = 0.006) lower risk of subsequent major VE. Furthermore, detectable viraemia at enrolment was associated with a 3.6 (95%CI 1.94, 6.70, *p*<0.001) times increased risk of subsequent major VE; CD4+> 500 cells/mm was associated with a 53% lower risk of major VE risk (95%CI 0.32, 0.89, *p* = 0.016).

**Table 2:** Factors associated with the risk of major viraemic episodes (viral load >1000 copies/ml) after achieving viral suppression: univariable and multivariable mixed‐effects Poisson regression model results expressed as incidence rate ratios (IRRs) with 95% confidence intervals (CIs)

	Univariable analysis			Multivariable analysis		
Characteristic	IRR	95% CI	*p*‐value	IRR	95% CI	*p*‐value
**Age (years)**	0.92	0.87, 0.96	<0.001	0.94	0.89, 0.98	0.006
**Gestational age**						
< 14 weeks	—	—		—	—	
> 14 weeks	0.91	0.49, 1.68	0.8	0.94	0.59, 1.52	0.8
**Gravidity**						
Primigravida	—	—		—	—	
Multigravida	0.61	0.29, 1.25	0.2	1.04	0.55, 1.95	0.9
**ART status**						
Continuers	—	—		—	—	
Initiators	3.22	1.40, 7.39	0.006	1.12	0.52, 2.37	0.8
Switcher	4.76	2.42, 9.37	<0.001	1.96	1.07, 3.58	0.03
**Viral load at enrolment (copies/ml)**						
<50	—	—		—	—	
50−1000	7.22	3.92, 13.3	<0.001	3.60	1.94, 6.70	<0.001
>1000	7.88	3.35, 18.6	<0.001	3.60	1.57, 8.28	0.003
**CD4+ count at enrolment (cells/mm^3^)**						
< 500	—	—		—	—	
> 500	0.33	0.19, 0.60	<0.001	0.53	0.32, 0.89	0.016
**Education**						
Any basic education	—	—		—	—	
Any post secondary	0.86	0.23, 3.21	0.8	0.71	0.25, 2.01	0.5

Abbreviations: ART, antiretroviral therapy; CI, confidence interval; IRR, incidence rate ratio.

## 4 DISCUSSION

These are among the first data describing the patterns of VS during pregnancy and the postpartum period among WHIV on TLD in public‐sector services in South Africa. Almost all WHIV had at least one VL <50 copies/ml, and 90% had an undetectable VL through pregnancy and the early postpartum period, the threshold below which the risk of perinatal transmission approaches zero. However, by 1 year postpartum, 15% of WHIV had at least one major VE (>1000 copies/ml), and VL was unsuppressed in one in five WHIV at 24 and 48 weeks postpartum. The high rate of VS in this cohort of pregnant and postpartum WHIV on TLD is consistent with published findings in non‐pregnant adults and clinical trial results in pregnant/postpartum people [3, 4, 5, 6, 7, 8, 22, 23]. Nonetheless, despite TLD's superior potency and excellent tolerability [24, 25], pregnant and postpartum WHIV continue to experience VEs, which occur with increasing frequency over time and have important implications for maternal health, as well as efforts to prevent new paediatric acquisitions.

At enrolment into ANC, close to 90% of women were already on ART, including 74% on TLD. This high proportion of women on ART at ANC entry is consistent with global trends [26, 27, 28]. Historically, most women learned their HIV status and initiated ART upon entry in ANC; however, with the successful scale‐up of HIV treatment programmes in sub‐Saharan Africa, many countries, including South Africa [10], are reporting that the majority of pregnant WHIV are entering ANC on treatment [29]. Nonetheless, despite the transition to TLD, 26% of WHIV on ART were still receiving TLE at enrolment. Others have also reported delayed TLD rollout for WHIV, a legacy of the repudiated concerns about an association between in utero DTG exposure and neural tube defects [22, 30, 31, 32]. Furthermore, we note that among those on ART, those on TLD at enrolment had markedly higher rates of VS compared to those on TLE, 89% versus 49%. We hypothesize that lower rates of VS among WHIV on TLE likely reflect differences in regimen efficacy, as well as adherence and care‐seeking behaviours among those who had not been switched to TLD. As VL is not routinely measured at entry into public sector ANC, these are among the first programme data to demonstrate high rates of VS among pregnant WHIV receiving TLD.

Overall, 99% of WHIV included in the analysis had at least one VL <50 cps/ml, that is achieved full VS at some point, and three‐quarters maintained VS across all time points. VEs were infrequent during pregnancy and the early postpartum period, but at 24 and 48 weeks, only 80% of WHIV maintained a suppressed VL. In our previous study from the same setting of women who initiated TLE in pregnancy and achieved VS, 22% had a major VE postpartum [16]. These findings suggest that adherence attrition is the primary cause of VE in this population [33]. The postpartum period has been identified as a particularly vulnerable time when WHIV may be at increased risk for poor adherence. The added demands of caring for an infant, transitions out of integrated HIV/ANC services to routine or differentiated ART services postpartum and low literacy regarding how ART reduces the risk of postnatal transmission have been associated with waning adherence postpartum [16, 29, 34, 35, 36, 37]. While it is likely that these factors are relevant to the women in this cohort, it is also possible that adherence lapsed among WHIV after discontinuing breastfeeding (median duration 19 weeks) when the motivation to protect their child from new HIV acquisitions ended.

We found several factors to be associated with the risk of major VEs, including younger age and viraemia at enrolment. WHIV with VS at enrolment were significantly more likely to remain suppressed, while each additional year of age conferred a 6% lower risk of a subsequent major VE. At the same time, WHIV in the ART Initiator sub‐cohort were significantly younger than both WHIV in the Continuers and Switchers groups, and as expected, less likely to be virally suppressed at enrolment. WHIV in the Initiators group generally started ART within weeks of enrolment, having newly tested positive on entry into ANC. Multiple studies have identified adolescent and young WHIV to be at higher risk for ART adherence challenges as well as poor maternal and child health outcomes [10, 38].

WHIV on TLE at enrolment, those switching to TLD, were a particularly interesting group. They appear similar to Continuers in terms of age and parity, but CD4+, VL and duration of breastfeeding were more similar to Initiators. In particular, we noted a higher rate of viraemia among WHIV in the Switcher group compared to WHIV continuing TLD at enrolment. It is possible that WHIV who were not switched prior to pregnancy were having adherence or retention challenges that lead to delays in the decision to transition to TLD. On the other hand, some WHIV in the Switcher group may have been on multi‐month dispensing schedules or other differentiated service delivery models that included less frequent interactions with healthcare providers, leading to delays in regimen transition. Further research is warranted to understand if the observed patterns are a unique feature of this cohort or a more generalizable phenomenon.

Our findings may not be broadly generalizable given the characteristics of our study population and the setting where the study was conducted. Pregnant WHIV were enrolled into the ORCHID cohort early in pregnancy, up to 18 weeks of gestation. The cohort did not include WHIV with late or no ANC who are historically at the highest risk for postpartum viraemia [16]. If they had been included, it is likely that rates of VS would have been lower and VEs more common. In addition, we excluded 41 WHIV (5% of the cohort) who were lost‐to‐study follow‐up and had only a single VL measure; these women likely constitute a population at higher risk for poor adherence and VEs. Furthermore, the cohort was recruited in the Western Cape, South Africa, a setting with high ART coverage, integrated ANC/HIV services, and high uptake of ANC services and facility‐based deliveries. Results may differ in settings with less robust health systems and populations with different healthcare‐seeking behaviours. In the Western Cape, WHIV transition out of integrated HIV/ANC services to routine ART services after delivery, whereas many countries recommend integrated maternal/child follow‐up services where attention to the needs of new mothers may be greater [39, 40]. Also, breastfeeding duration among WHIV in our population was relatively short compared with other settings in South Africa and elsewhere in sub‐Saharan Africa [41]. The short duration of breastfeeding reported among WHIV in this cohort is similar to what we reported in a prior study, approximately 16 weeks, conducted in the same setting, a decade ago, among pregnant WHIV initiating TLE [39].

## 5 CONCLUSIONS

Despite optimization of ART regimens and HIV service delivery, sustaining VS remains a challenge for many individuals, particularly WHIV during the postpartum period. WHIV in the ORCHID cohort achieved high rates of VS during pregnancy and the early postpartum period, an important finding in our efforts to prevent new paediatric acquisitions. Unfortunately, one of every five WHIV failed to maintain VS and had evidence of viraemia by 24 weeks after delivery. These data will inform global guidance for vertical transmission prevention, including VL monitoring and postnatal infant prophylaxis. Furthermore, these observations provide further impetus to explore innovative approaches to supporting adherence among WHIV during periods of vulnerability, particularly during the postpartum period, including the role of long‐acting ART and differentiated models of care to ensure optimal maternal and child health outcomes [10, 39, 42, 43].

## COMPETING INTERESTS

The authors have declared that no competing interests exist.

## AUTHOR CONTRIBUTIONS

This analysis was conceptualized by EJA, JJ and LM with EJA, EM and LM conducting the data analysis and interpretation. SM, JL, HPM and AZ oversaw project administration. EJA, EM and LM drafted the original version of the manuscript, and SM, JJ, HPM, JL and AZ provided manuscript review and edits. All authors have read and approved the final manuscript.

## FUNDING

This work is supported by the Eunice Kennedy Shriver National Institute of Child Health & Human Development (NICHD) of the National Institutes of Health [R01HD104599]. The funder had no role in study design, data collection and analysis, decision to publish, or preparation of the manuscript.

## DISCLAIMER

The content is solely the responsibility of the authors and does not necessarily represent the official views of the National Institutes of Health.

## Data Availability

After completion of the ORCHID study, de‐identified datasets will be prepared and made available to the NICHD Data and Specimen Hub (DASH), a centralized resource for researchers to store de‐identified data and to access data and associated biospecimens from NICHD‐supported studies for use in secondary research.
